# Stable transmission of complex chromosomal rearrangements involving chromosome 1q derived from constitutional chromoanagenesis

**DOI:** 10.1186/s13039-019-0455-z

**Published:** 2019-10-31

**Authors:** Mary A. Gudipati, Elizabeth Waters, Carol Greene, Nidhi Goel, Nicole L. Hoppman, Beth A. Pitel, Matthew R. Webley, Ying Zou

**Affiliations:** 1 Department of Pathology, University of Maryland School of Medicine, Baltimore, MD USA; 2 Department of Pediatrics, University of Maryland School of Medicine, Baltimore, MD USA; 3 Department of Internal Medicine, University of Maryland School of Medicine, Baltimore, MD USA; 4 Laboratory Medicine and Pathology, Mayo Clinic, Rochester, MN USA; 5 Department of Pathology, Johns Hopkins University, 1812 Ashland Ave., Suite 200, Room 221, Baltimore, MD 2120 USA

**Keywords:** Chromoanagenesis, Chromothripsis, Chromoanasynthesis, Chromoplexy, Constitutional 1q abnormalities

## Abstract

**Background:**

Chromoanagenesis events encompassing chromoanasynthesis, chromoplexy, and chromothripsis are described in cancers and can result in highly complex chromosomal rearrangements derived from ‘all-at-once’ catastrophic cellular events. The complexity of these rearrangements and the original descriptions in cancer cells initially led to the assumption that it was an acquired anomaly. While rare, these phenomena involving chromosome 1 have been reported a few individuals in a constitutional setting.

**Case presentation:**

Here, we describe a newborn baby who was initially referred for cytogenetic testing for multiple congenital anomalies including cystic encephalomalacia, patent ductus arteriosus, inguinal hernia, and bilateral undescended testicles. Chromosome analysis was performed and revealed a derivative chromosome 1 with an 1q24-q31 segment inserted into 1q42.13 resulting in gain of 1q24-q31. Whole genome SNP microarray analysis showed a complex pattern of copy number variants with four gains and one loss involving 1q24-q31. Mate pair next-generation sequencing analysis revealed 18 chromosome breakpoints, six gains along an 1q24-q31 segment, one deletion of 1q31.3 segment and one deletion of 1q42.13 segment, which is strongly evocative of a chromoanasynthesis event for developing this complex rearrangement. Parental chromosome analyses were performed and showed the same derivative chromosome 1 in the mother.

**Conclusions:**

To our knowledge, our case is the first case with familial constitutional chromoanagenesis involving chromosome 1q24-q42. This report emphasizes the value of performing microarray and mate pair next-generation sequencing analysis for individuals with germline abnormal or complex chromosome rearrangements.

## Background

Chromoanagenesis events are new types of complex and massive chromosomal and genomic alterations characterized by the simultaneous occurrence of multiple structural rearrangements confined to one or a few chromosomal segments through a single catastrophic cellular event [1–3]. The term ‘chromoanagenesis’ was used to describe a new ‘all-at-once’ process, identified by genome sequencing techniques and bioinformatics tools as a new driver of tumorigenesis by which, challenging the well-known mechanism of gradual accumulation of mutations to prefer cell duplication/survival, a single catastrophic event of massive shattering and disordered reassembly of one or few chromosomes induced oncogenic lesions [3]. Therefore, the concept of chromoanagenesis, a form of chromosome rebirth, provides new insight into the nature of complex chromosomal rearrangements. Chromoanagenesis has been encompassed at least three phenomena independent of the underlying mechanism: the chromoplexy, the chromothripsis, and the chromoanasynthesis [1–3].

The chromoplexy is characterized by the interdependent occurrence of multiple inter-and intra-chromosomal translocations and deletions [4]. The chromothripsis is defined as a mutational event driven by multiple double-strand breaks occurring in a single catastrophic event between several chromosomes/segments and followed by NHEJ-mediated repair mechanisms (the reassembly of the DNA fragments in random order and orientation to form complex derivative chromosomes) with or without copy number changes [3]. Thus, the chromothripsis-related structural rearrangements usually include deletions, insertions and inversions. Being a chromosome shattering phenomenon, complex genomic rearrangements can occur at a part of or an entire chromosome, or few chromosomes. In contrast to the chromothripsis/chromoplexy, the chromoanasynthesis is a replication based complex rearrangement process that involves serial fork stalling and template switching or microhomology-mediated break-induced replication mechanisms [5–7], which can lead to a highly remodeled chromosomes with copy number changes including gains and losses along a single chromosome. Therefore, chromothripsis and chromoanasynthesis could frequently explain the formation of multiple copy number changes on the same chromosome.

The complexity of these rearrangements and the original descriptions in cancer cells initially led to the assumption that chromoanagenesis was an acquired anomaly [3]. While rare, chromoanagenesis-related complex chromosomal rearrangements involving chromosome 1 have been reported a few individuals in a constitutional setting [5, 8–13]. Majority of these complex chromosomal rearrangements involve translocations between chromosome 1 and other chromosomes instead of a single rearranged chromosome 1. Furthermore, not all breakpoints were well characterized at a high resolution. Here, we describe the first case of a newborn baby with multiple congenital anomalies and very complex chromosomal rearrangements involving a long arm of chromosome 1 (at 1q24-q42) inherited from his mother. The 18 breakpoints along the long arm of chromosome 1 of our patient were well characterized by whole genome SNP microarray and mate pair next-generation sequencing analyses, which provides new insight into the nature of a chromoanagenesis event.

## Case presentation

During the pregnancy of the proband by a 23-year-old G1P0 mother, prenatal ultrasound of the fetus revealed congenital anomalies including dilated right cerebral ventricle (suspected germinal matrix hemorrhage), pyelectasis, echogenic bowel, and hypoplastic nasal bone. 36-week gestational fetal MRI revealed cystic encephalomalacia in region of left caudate nucleus and caudothalamic groove appears more conspicuous, likely reflecting sequela of germinal matrix hemorrhage, similar prominent left lateral ventricles without evidence of obstructive hydrocephalus, and mild left fetal pyelocaliectasis.

The proband was born at 37-week-6-day gestational age by cesarean due to breech presentation, and abnormal prenatal ultrasound/MRI findings. The mother was group B streptococcus positive. At birth, his weight was 2.915 kg, his length is 47 cm, and his occipital–frontal circumference (OFC) was 34.5 cm. His Apgars were 7 and 8 at 1 and 5 min, respectively. He had decreased respiratory effort after birth and requiring blow by oxygen briefly. His echo showed small patent ductus arteriosus with left to right shunting, ductal velocity indicating elevated pulmonary artery pressures, and insufficient tricuspid valve regurgitation for estimation of right ventricular systolic pressure. His ultrasound revealed resolving left germinal matrix hemorrhage. He had no acute hemorrhage, no pyelectasis, no echogenic bowel, and a normal size of the ventricles. His MRI revealed periventricular white matter cystic encephalomalacic change at the left frontal horn and caudothalamic groove, likely representing sequela of prior germinal matrix hemorrhage. He had bilateral undescended testes with right testis located within the inguinal canal, and the left testis seen coursing between the left lower pelvis and upper inguinal canal.

At the age of 4 months, his height was 55.9 cm (<1st centile; 50th centile for a 1-month old), his weight was 5.1 kg (<1st centile; 50th centile for an 1.5-month old), and his OFC was 41.4 cm (38th centile). He had failure to thrive, developmental delay, severe tracheomalacia, stridor/difficulty breathing along with decreased oral intake, bilateral inguinal, gastroesophageal reflux disease, and bilaterally undescended testes. He also had possible seizure disorder, increased tone, and abnormal rigid movements with significant jitteriness and frequent myoclonic jerks. His anterior fontanelle was open and flat. He had facial dysmorphisms including small nose and depressed nasal bridge with possible hypertelorism.

His mother had intellectual disability and lived with her mother. Paternal grandmother and paternal great grandmother had seizure. Father’s family members had attention deficit hyperactivity disorder, anxiety, and depression disorders. No cancer-related disorders were found on either side of the family.

## Results

**Fig. 1:**
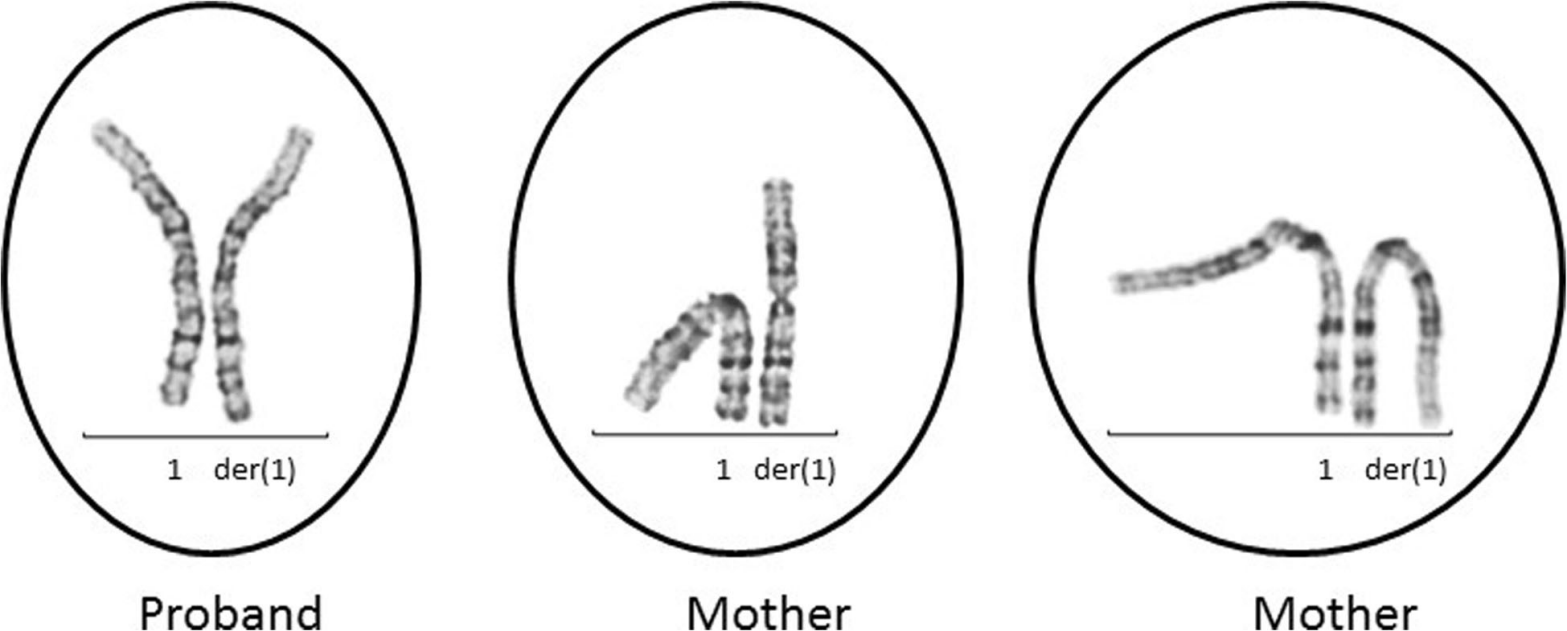
Partial karyogram showing abnormal derivative chromosome 1 from the proband and the mother

**Fig. 2:**
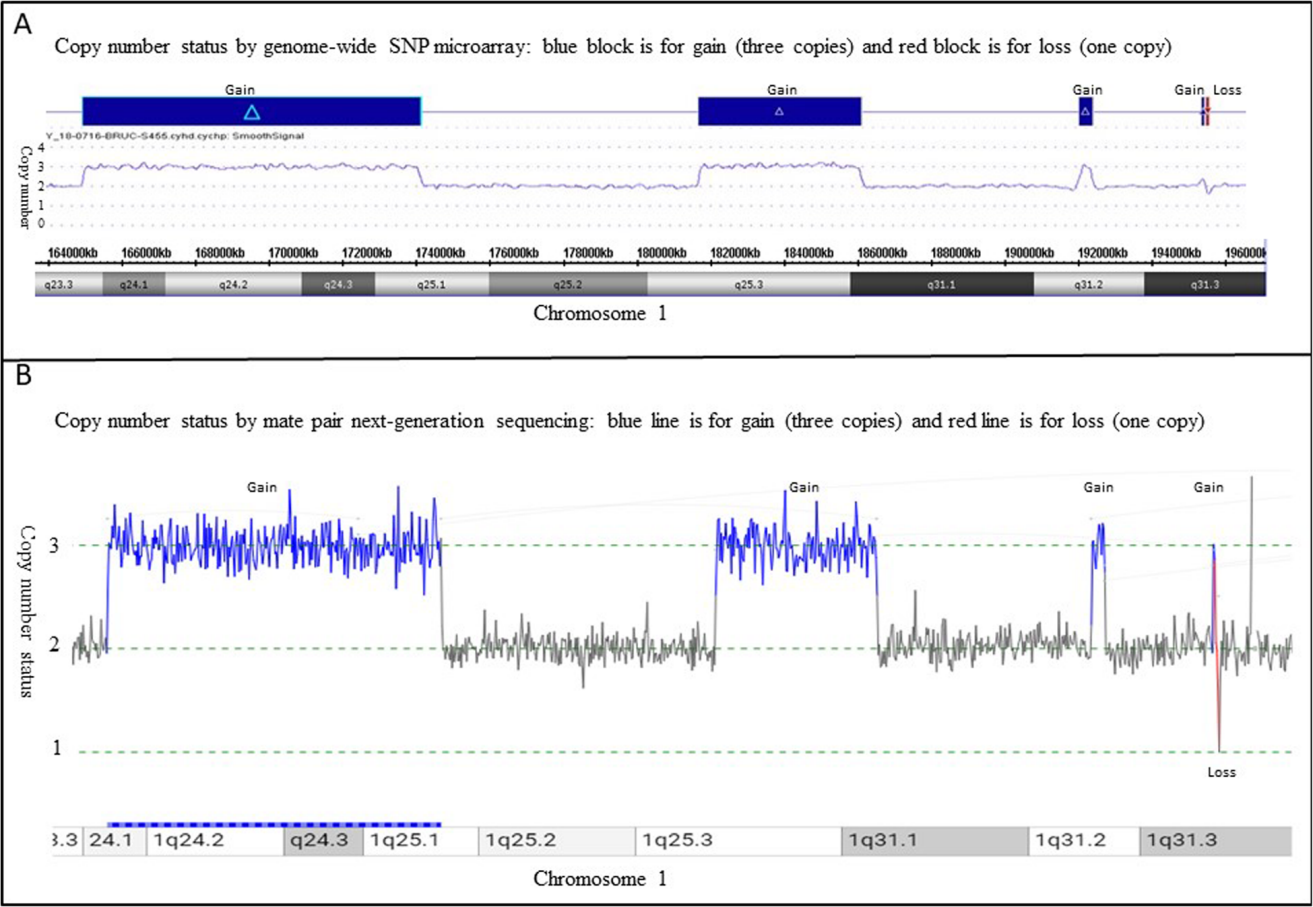
Copy number changes on the long arm of chromosome 1q24-q31 region. **a** Genome-wide SNP microarray revealed four gains (blue blocks) and one loss (red block), the X axis is the long arm of chromosome 1q23.3-1q31.3 region, the Y axis is for the copy number, blue and red blocks are for gain and loss of 1q, respectively; **b** Consistent with Genome-wide SNP microarray, mate pair next-generation sequencing also revealed four gains and one loss. Blue and red lines are for gain and loss of 1q, respectively

His blood karyotype is 46,XY,der(1)ins(1;1)(q42.13;q24q31), with interstitial gain of the 1q24-q31 segment (Fig. 1). Genome-wide SNP-microarray (CytoscanHD chip, Thermo Fisher Scientific using Chromosome Analysis Suite (ChAS) version 3.3 for the SNP analysis) demonstrates 1q24.1q25.1(166,124,606-175,038,371)× 3,1q25.3q31.1(182,388,825-186,693,330)× 3,1q31.2(192,446,379-192,799,227)× 3,1q31.3(195,686,410-195,745,969)× 3, 1q31.3(195,828,516-195,876,205)× 1, which are 8.9 Mb gain of 1q24.1-q25.1, 4.3 Mb gain of 1q25.3-q31.1, 352 Kb gain of 1q31.2, 60 Kb gain of 1q31.3 and 48 Kb loss of 1q31.3 (Fig. 2a). Therefore, the propositus had an unbalanced derivative chromosome 1 with four gains and one loss along the 1q24-q31 region (Fig. 2a). His father had a normal karyotype, while his mother carried the same derivative chromosome 1 as observed in her son (Fig. 1) and her CytoScanHD SNP microarray (Thermo Fisher Scientific, Waltham, MA) revealed the same gains and loss along the 1q24-q31 region as her son, further supporting that her son’s derivative chromosome 1 was inherited from the mother.

**Fig. 3:**
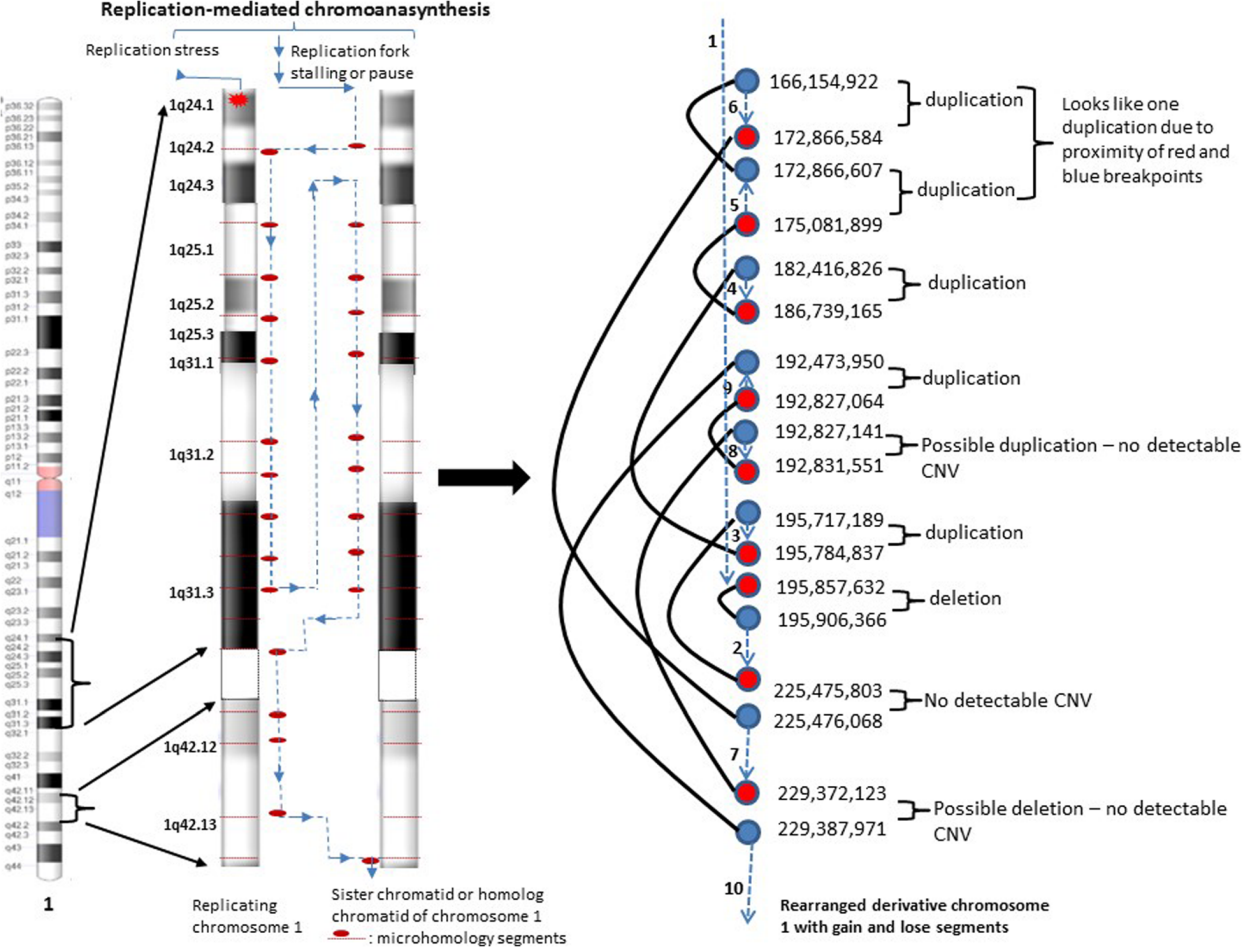
Characterization of the derivative chromosome 1 by mate pair next-generation sequencing. From left to right: ideogram of chromosome 1, replicating chromosome 1 showing 1q24-q42 region, sister chromatid/homolog chromatid of chromosome 1, and chromosome 1q24–42 had 18 breakpoints, nine chromosome junctions, six gains and two losses, suggesting replication-mediated chromoanasynthesis by fork stalling and template switching or microhomology-mediated break-induced replication as responsible to form a highly complex chromosome 1

To further characterize the derivative chromosome 1, mate pair next-generation sequencing [11, 12] of the proband was performed and revealed complex rearrangements involving chromosome 1q. The derivative chromosome 1 had complicated rearrangements with four gains and one deletion along the 1q24-q31 region (Fig. 2b) and 18 breakpoints along 1q24–31 and 1q42 regions, seq [GRCh38] inv.(1)(pter- > q31.3(195,857, 632)::q31.3(195,906,366)- > q42.12(225,475,803)::q31.3(195,717,189)- > q31.3(195, 784,837)::q25.3(182,416,826)- > q31.1 (186,739,165)::q25.1(175,081,899)- > q24.3(172,866,607)::q24.1(166,154,922)- > q24.3(172,866,584)::q42.12(225,476,068)- > q42.13(229,372,123)::q31.2(192,827,141)- > q31.2(192,831,551)::q31.2(192,827,064)- > q31.2(192,473,950)::q42.13(229,387,971)- > qter), resulting in six gains and two losses along 1q (Fig. 3).

## Discussion and conclusions

Chromoanagenesis events can lead to complex and massive chromosomal rearrangements and contains chromoplexy, chromothripsis, and chromoanasynthesis events [2]. The chromoplexy can frequently create chromosomal translocations and deletions [4]. The chromothripsis usually produces deletions, insertions and inversions. Deletions are generally more common than gains of genetic material through a typical chromothripsis event. In contrast to the chromothripsis / chromoplexy, the chromoanasynthesis can typically lead to a highly remodeled chromosomes with copy number changes along a single chromosome [5–7]. Gains are more common than deletions in a usual chromoanasynthesis event. Our patient had multiple gains and losses clustered on a single chromosome arm (the long arm of chromosome 1), which support the complex 1q rearrangement in our patient may arise through either a chromothripsis or a chromoanasynthesis event. Since the complex 1q rearrangements in our patient had more gains (six gains) than deletions (two losses), which may support the presence of a chromoanasynthesis event as responsible to form a highly complex rearrangement of chromosome 1 with copy number changes by fork stalling and template switching and microhomology-mediated break-induced replication (Fig. 3).

Patients with chromoanasynthesis-mediated rearrangements have been reported to display developmental delay, intellectual disability, dysmorphic features, or relatively mild phenotypic effects [1, 14], and the severity of clinical presentation will depend on dosage-sensitive genes located in these gain and loss regions. Our patient had six gains and two losses at the highly rearranged 1q (Table 1). Although no genes locate at one loss and two gains, the four gains in our patient contain ~ 258 RefSeq genes, 89 OMIM genes, and 20 disease genes (Table 1). Among eight autosomal dominant disease genes, he PRRX1 gene (OMIM: 167420) encodes a homeobox gene, expresses in specific temporal and spatial patterns, functions as transcriptional regulators of developmental processes [15], and has important roles during the patterning of the first pharyngeal arch and mandibulofacial development [16]. Heterozygous loss-of-function mutation in the PRRX1 gene can lead to agnathia-otocephaly complex (OMIM: 202650), which is a rare and lethal condition characterized by mandibular hypoplasia or agnathia, ventromedial auricular malposition (melotia) and/or auricular fusion (synotia), microstomia with oroglossal hypoplasia or aglossia, holoprosencephaly, and skeletal, genitourinary, and cardiovascular anomalies [17]. Gain of the PRRX1 gene has been reported in patients with intellectual disability, global developmental delay, seizures, autistic behavior, plagiocephaly, cardiomyopathy, and facial dysmorphism (DECIPHER patients 2365, 264469, 277957, 285697, 285898, 293723, 317779, 332725). The gains in our patient also contain two autosomal dominant disease genes associated with eye disorders, heterozygous mutation in the MYOC (OMIM: 601652) and the HMCN1 (OMIM: 608548) genes have been associated with one form of primary open angle glaucoma 1A (OMIM: 137750) and susceptibility to age-related macular degeneration-1 (OMIM: 603075), respectively. Smaller gains as our patients have also been reported in patients with intellectual disability, global developmental delay, autistic behavior, and facial dysmorphism (DECIPHER patients 273428, 293641, 343360, 362331, and ClinVar patient nsv530079, etc.).

**Table 1:** Genes locate at six gains and two losses of the rearranged 1q in our patient

Start site (bp)	End site (bp)	Size (Kb)	RefSeq genes	OMIM genes	Disease genes	Autosomal recessive disease genes	Autosomal dominant disease genes
Six gains							
166,154,922	172,866,584	6712	132	45	12	CD247, TBX19, SLC19A2, F5, GORAB, PRRX1, FMO3, PIGC	ADCY10, F5, PRRX1, MYOC, EEF1AKNMT, FASLG
172,866,607	175,081,899	2215	51	14	3	DARS2, SERPINC1, MRPS14	SERPINC1
182,416,826	186,739,165	4322	68	27	5	LAMC2, NCF2, TSEN15, PRG4	HMCN1
192,473,950	192,827,064	353	7	3	0		
192,827,141	192,831,551	4	0	0	0		
195,717,189	195,784,837	68	0	0	0		
Two losses							
195,857,632	195,906,366	49	0	0	0		
229,372,123	229,387,971	16	1	0	0		

Beside six gains and two losses, our patient had 18 breakpoints and nine chromosome junctions (Table 2). Two breakpoints locate at introns of two genes, FAM78B and TNN (OMIM: 617472). The FAM78B gene is novel with unknown function. It is 109 Kb in size, has 261 amino acids, codes two exons, and makes a 29.8 KDa protein [18]. The TNN gene encodes an extracellular matrix glycoprotein with a characteristic structure consisting of an N-terminal cysteine-rich segment, EGF-like repeats, fibronectin type III repeats, and C-terminal fibrinogen-like domain [19]. The TNN gene expresses in all brain regions, with a graded staining pattern in the hippocampal CA3 region and may associate with cell migration and neurite growth [19]. It has been associated with low bone mineral density and primary myopathies [20, 21]. While our patient had no genes located at one loss, two gains and 16 breakpoints of the abnormal chromosome 1 (Table 2), it is possible that positional effects may influence the expression of nearby dosage-sensitive genes, contributing to abnormal phenotype.

**Table 2:** DNA sequences flanking the 18 breakpoints in our patient

	Breakpoints at Chromosome 1	Repeat Family	Repeat Class	Genes
1	192,827,141	L1	LINE	
2	195,857,632	L1	LINE	
3	172,866,584	L2	LINE	
4	172,866,607	L2	LINE	
5	192,473,950	L2	LINE	
6	192,831,551	CR1	LINE	
1	225,475,803	Alu	SINE	
2	225,476,068	Alu	SINE	
3	229,387,971	Alu	SINE	
4	166,154,922	MIR	SINE	FAM78B
5	186,739,165	MIR	SINE	
1	175,081,899	ERV1	LTR	TNN
2	229,372,123	ERV1	LTR	
3	182,416,826	ERVL	LTR	
4	195,906,366	ERVL	LTR	
1	195,717,189	Simple repeat	Simple repeat	
2	195,784,837	Simple repeat	Simple repeat	
1	192,827,064	No known repeat	No known repeat	

In order to understand genome architecture at rearrangements’ breakpoints and role of unusual DNA sequences such as low-copy repeats or tandem repeats in chromoanagenesis, we checked for all repeat elements at the distal and proximal sites of the 18 breakpoints using RepeatMasker [http://www.repeatmasker.org] and Repbase update programs [22]. We detected a variety of repeats at 17 out of 18 breakpoints, which include long interspersed nuclear elements (LINE, a total of 6), short interspersed nuclear elements (SINE, a total of 5), long terminal repeat elements (LTR, a total of 4), and other simple repeat elements (a total of 2) (Table 2). These repetitive sequences create points of genomic instability and may serve as substrates for chromosomal rearrangements [23, 24]. Our patient had LINE sequences and *Alu* repeats at breakpoints (Table 2). LINE-1 s (L1 s) are endogenous mutagens and have both DNA endonuclease [25] and reverse-transcriptase activities [26]. L1 is capable of mobilizing itself [27, 28] and other retrotransposons such as *Alu* [29, 30]. There is also a correlation between retrotransposon sequences and genomic structural variants [31–34] and segmental duplications [35]. In particular, L1-mediated retrotransposition and homologous recombination between *Alu* repeats may serve as potential mutagens in the genome [36]. The abundance of these elements at breakpoints in our patient may suggest an association of active and inactive retrotransposons at a chromoanagenesis event. Further studies of other breakpoint junctions involved in constitutional chromoanagenesis cases will be necessary to elucidate the role of these endogenous mutagens in chromoanagenesis formation.

To our knowledge, our case is the first case with familial constitutional chromoanagenesis involving chromosome 1q24-q42. Constitutional chromoanagenesis have likely been underestimated in a constitutional setting. Microarray and mate pair next-generation sequencing technologies can be used to accurately detect such complexity. Further characterization of these breakpoint junctions in our patient will help understand the molecular mechanisms responsible for this process of massive genomic rearrangement of chromosome 1q.

## Data Availability

The data sets used and/or analyzed during the current study are available from the corresponding author on reasonable request. All authors read and approved the final manuscript.

## Abbreviations

1q: The long arm of chromosome 1
cm: Centimeter
Kg: Kilogram
MRI: Magnetic resonance imaging
NHEJ: Non-homologous end joining
OFC: Occipital–frontal circumference
OMIM: Online Mendelian Inheritance in Man
SNP: Single nucleotide polymorphism
